# Efficacy and safety of electroacupuncture in acute decompensated heart failure: a study protocol for a randomized, patient- and assessor-blinded, sham controlled trial

**DOI:** 10.1186/s12906-017-1864-3

**Published:** 2017-07-11

**Authors:** Jungtae Leem, Seung Min Kathy Lee, Jun Hyeong Park, Suji Lee, Hyemoon Chung, Jung Myung Lee, Weon Kim, Sanghoon Lee, Jong Shin Woo

**Affiliations:** 1 DongShin Korean Medicine Hospital, 351, Omok-ro, Yangcheon-gu, Seoul, 07999 Republic of Korea; 2 ChungYeon Medical Institute, 64, Sangmujungang-ro, Seo-gu, Gwangju, 61949 Republic of Korea; 3 Department of Clinical Korean Medicine, Graduate School, Kyung Hee University, 26 Kyungheedae-ro, Dongdaemun-gu, Seoul, 02447 Republic of Korea; 4 Department of Acupuncture and Moxibustion, Kyung Hee University Korean Medicine Hospital, 26 Kyungheedae-ro, Dongdaemun-gu, Seoul, 02447 Republic of Korea; 5 Division of Cardiology, Department of Internal Medicine, Kyung Hee University Hospital, Kyung Hee University, 26 Kyungheedae-ro, Dongdaemun-gu, Seoul, 02447 Republic of Korea; 6 Department of Medicine, Graduate School, Kyung Hee University, 26 Kyungheedae-ro, Dongdaemun-gu, Seoul, 02447 Republic of Korea

**Keywords:** Heart failure, Electroacupuncture, Acupuncture, Randomized controlled trial

## Abstract

**Background:**

The purpose of this trial is to evaluate the effectiveness and safety of electroacupuncture in the treatment of acute decompensated heart failure compared with sham electroacupuncture.

**Methods:**

This protocol is for a randomized, sham controlled, patient- and assessor-blinded, parallel group, single center clinical trial that can overcome the limitations of previous trials examining acupuncture and heart failure. Forty-four acute decompensated heart failure patients admitted to the cardiology ward will be randomly assigned into the electroacupuncture treatment group (*n* = 22) or the sham electroacupuncture control group (*n* = 22). Participants will receive electroacupuncture treatment for 5 days of their hospital stay. The primary outcome of this study is the difference in total diuretic dose between the two groups during hospitalization. On the day of discharge, follow-up heart rate variability, routine blood tests, cardiac biomarkers, high-sensitivity C-reactive protein (hs-CRP) level, and N-terminal pro b-type natriuretic peptide (NT-pro BNP) level will be assessed. Four weeks after discharge, hs-CRP, NT-pro BNP, heart failure symptoms, quality of life, and a pattern identification questionnaire will be used for follow-up analysis. Six months after discharge, major cardiac adverse events and cardiac function measured by echocardiography will be assessed. Adverse events will be recorded during every visit.

**Discussion:**

The result of this clinical trial will offer evidence of the effectiveness and safety of electroacupuncture for acute decompensated heart failure.

**Trial registration:**

Clinical Research Information Service: KCT0002249.

## Background

Heart failure (HF) is defined as *“a complex clinical syndrome that can result from any structural or functional cardiac disorder that impairs the ability of the ventricle to fill or eject blood.”* according to the American Heart Association guidelines [1]. Conventional Western medical treatment for HF includes beta blockers, angiotensin-converting enzyme inhibitors (or angiotensin receptor blockers), and diuretics [2]. The overall prevalence of HF is estimated to be from 1% to 12%; it is known that there are more than 23 million HF patients worldwide [3]. Therapeutic improvement for HF and an aging population has led to increased prevalence of HF. In spite of therapeutic developments for HF, mortality is still high [4]. Due to chronic clinical course and frequent readmission, the burden of HF is increasing [3]. Further, despite therapeutic improvements, the 5-year survival rate of HF is 50%, and the 10-yr survival rate is only 10% [5, 6]. Therefore, another treatment approach is needed, and traditional East Asian therapies such as acupuncture and herbal medicine may be considered in this regard [7–10].

One of the important pathophysiologies of HF is autonomic nervous system (ANS) imbalance [11]. It is known that heart rate variability (HRV) reflects ANS status [12]. HRV predicts the mortality and prognosis of HF patients [13, 14]. Acupuncture is known to modify the ANS tone [12], and it is already known that several acupuncture points, such as PC6 and ST36, affect ANS tone through regulation of sympathetic outflow from rostral ventral medulla in the central autonomic pathway [15, 16]. Several acupuncture clinical trials have been conducted [17–23]. However, according to a recent systematic review of acupuncture treatment for HF, the studies had several limitations and the methodological quality was relatively low [24]. The review suggested several implications for future clinical trials. It also suggested that several important outcomes should be included in future acupuncture clinical trials of HF. Clinical outcome variables such as mortality, major adverse cardiac events (MACE), and the New York Heart Association (NYHA) Functional Classification should be investigated. Other objective surrogate outcomes such as cardiac biomarkers, N-terminal pro b-type natriuretic peptide (NT-pro BNP), and cardiac function measured by echocardiography should also be included. Additionally, total diuretic doses for each group during hospitalization should be reported, as excessive diuretics cause an electrolyte imbalance that increases the risk of a cardiovascular event. In terms of intervention, most HF acupuncture experimental studies used electroacupuncture, but only one clinical trial adopted electroacupuncture; the review suggested usage of electroacupuncture with a 2-Hz frequency. For the clinical trial process, the review suggested a long-term follow-up duration to assess the long-term effects of acupuncture treatment [24].

According to the implications for future clinical trials of the previous review, our study team planned an electroacupuncture clinical trial for acute decompensated HF patients that could overcome previous limitations. First, our primary outcome was the total diuretic dose administered during hospitalization, which has been recorded in several acute HF clinical trials [25–28]. As acupuncture treatment is an adjunctive treatment in cases of HF, our research team concluded that reducing the total dose and the adverse effects of the diuretics through acupuncture treatment is meaningful. Second, we included several important outcomes, such as mortality, MACE, NYHA Functional Classification, cardiac biomarkers, NT-pro BNP, and cardiac function measured by echocardiography. Third, we adapted the electroacupuncture protocol. In addition, we adapted a Park Sham Device (PSD; Acuprime, Exeter, UK), a validated sham acupuncture device [29], to maintain blindness and non-biased measurement of subjective outcomes. Last, we will follow the participants until 6 months after discharge to assess the long-term effects of acupuncture treatment in acute HF.

## Methods/design

Our protocol was written in accordance with Standard Protocol Items: Recommendations for Interventional Trials (SPIRIT) [30] which was aim to improve the quality of clinical trial.

### Objectives

The objective of this trial is to determine the effectiveness and safety of adjunctive electroacupuncture treatment administered in conjunction with conventional treatments in cases of acute decompensated HF patients, as measured by diuretic dose, electrolyte imbalance, renal function, HF symptoms, quality of life, cardiac biomarkers, NT-pro BNP, high-sensitivity C-reactive protein (hs-CRP), cardiac function, HRV, and MACE compared with sham electroacupuncture.

### Trial design and study setting

This study is a randomized, sham controlled, parallel group, patient- and assessor-blinded, single center clinical trial.

### Recruitment period and method

We will recruit participants who are admitted to the cardiology ward of Kyung Hee Medical Center (KHMC) for treatment of acute HF. Attending physicians (Western medicine cardiologists) will check the inclusion criteria and inform patients or guardians about the acupuncture clinical trial. If the patient or guardian agrees to participate in the trial, written informed consent will be obtained by attending physicians. Recruitment is expected to continue from March 2016 until May 2019. We will not use other advertising methods such as a homepage, bulletin boards, or mass media.

### Type of participants: inclusion and exclusion criteria

#### Inclusion criteria

Participants who satisfy the following criteria will be recruited:

1. Over 40 years of age, with written informed consent.
2. Acute HF patient presenting with dyspnea related to cardiogenic pulmonary edema and in need of diuretic treatment.
3. Systolic blood pressure > 95 mmHg at admission.
4. B-type natriuretic peptide (BNP) > 150 pg/mL or NT-pro BNP > 600 pg/mL.

#### Exclusion criteria

Participants who have any of the following conditions will be excluded from the study:

1. Body temperature > 38.0 °C or in need of intravenous antibiotic treatment due to acute inflammation or sepsis.
2. K < 3.5 mEq/L (patients with potassium levels of 3.0 to approximately 3.5 mEq/L may be admitted if they agree to receive potassium treatment).
3. Currently take or plan to take vasopressors, inotropics, have an intra-aortic balloon pump, extracorporeal membrane oxygenation, or endotracheal intubation.
4. Require a heart transplant
5. Present with possible acute coronary syndrome within 2 weeks
6. Acute HF related to significant arrhythmia (ventricular fibrillation, ventricular tachycardia, atrial fibrillation, atrial flutter, complete AV block)
7. Acute HF related to acute myocarditis, hypertrophic cardiomyopathy, restrictive cardiomyopathy, constrictive cardiomyopathy
8. In need of or currently receiving hemodialysis or peritoneal dialysis
9. Abnormal liver function (total bilirubin >3 mg/dL, albumin <2.8 mg/dL)
10. In need of a blood transfusion (hemoglobin <8 g/dL or hematocrit <25%)
11. Chronic obstructive pulmonary disease
12. Currently undergoing treatment for or with a history of malignant tumor
13. Pregnant or breast-feeding
14. Unable to follow up (outpatient clinic) for more than 6 months
15. Currently participating in another clinical trial.

### Randomization and allocation concealment

A random number table will be generated with the computerized random number generator Package randomizeR (R Core Team (2013), R Foundation for Statistical Computing, Vienna, Austria). The random number table will be prepared by an independent researcher. The random number file will be protected by a password. Allocation will be concealed until informed consent is obtained. If the participants satisfy the inclusion criteria and sign informed consent, the Korean Medicine Doctor who will conduct the acupuncture treatment will be informed of the group allocation.

### Blinding

The clinical research coordinator, assessor, attending physician, and participants will be blinded. Due to characteristics of the acupuncture clinical trial, the acupuncture practitioner cannot be blinded. To blind participants, we will use PSD regardless of group allocation. PSD is a validated sham device for acupuncture trials [29]. With the exception of PSD, other factors such as doctor-patient relationship, the clinical trial process, and the environment will be identical so that participants cannot guess their allocated group. The clinical research coordinator and the attending physician (a Western medicine cardiologist) will be blinded until completion of the study. The assessor will also be blinded. To maintain blinding, the assessor and participants will be educated according to the Standard Operation Procedure (SOP). We will notify allocated group to participants if serious adverse events occurs according to SOP.

To identify blinding success, direct assessment of blinding by the blinding index (BI) [31, 32] and indirect assessment by a credibility test [33] and acupuncture expectancy scale [34] will be conducted on Day 1 and Day 5 of the clinical trial. In the BI, patients will be required to select one of the following three statements: “I think I belong to the treatment intervention group”, “I think I belong to the control intervention group”, or “I do not know”. On the acupuncture expectancy scale, patients will answer the following four questions using a five-point Likert scale: *“ 1) My illness will improve a lot, 2) I will be able to cope with my illness better 3) The symptoms of my illness will disapper 4) My energy level will increase”*. In the credibility test, patients will answer the following four questions using a seven-point Likert scale:*“1) How confident do you feel that this treatment can alleviate your acute HF?”; “2) How confident would you be in recommending this treatment to a friend who suffered from a similar complaint?”; “ 3) How logical does this treatment seem to you?”; “ 4) In your opinion, how successful may this treatment be in alleviating other complaints?”*

### Intervention

#### Study flow

If the participants voluntarily sign informed consent during admission to the cardiology ward, participants will be screened according to inclusion and exclusion criteria on the screening day. After the subject fulfills the criteria, the basic characteristics and prognostic factors of each participant will be evaluated. The subjects will be randomized into an intervention and a control group with a 1:1 allocation ratio on the same day; the screening day and Day 1 will usually be the same day. From Day 1 to Day 5, the participants will receive electroacupuncture treatment or sham electroacupuncture treatment once a day for consecutive 5 days according to their allocated group. After discharge, participants will visit KHMC three times. Visit (V1) will be 7 days after discharge. Visit 2 (V2) will be 28 days after discharge. Visit 3 (V3) will be 180 days after discharge. On the day discharge (Day 5) and V2, blood tests, quality of life (QOL), and a pattern identification questionnaire according to traditional East Asian medicine (TEAM) theory will be evaluated. On V3, cardiac function and MACE will be evaluated by echocardiography (Table 1). Participants will be withdrawn from the study if they 1) re-admit to hospital related to cardiac or kidney dysfunction 2) withdraw of consent by participants 3) worsening cardiac function 4) the principle investigator judged that participants can not continue the research.

**Table 1:** Study schedule

Study period	Screening visit	Acupuncture treatment period					Follow-up period		
Visit	Screening	D1	D2	D3	D4	D5	V1	V2	V3
Informed consent	X								
Demographics and history collection	X								
Inclusion and exclusion criteria	X								
Randomization		X							
Physical examination and vital signs check	X	X	X	X	X	X	X	X	X
Body weight	X	X	X	X	X	X	X	X	X
Symptoms check (NYHA Functional Class)	X	X	X	X	X	X	X	X	X
Adverse events	X	X	X	X	X	X	X	X	X
Concomitant medication check	X	X	X	X	X	X	X	X	X
Routine blood test^a^	X		X			X		X	X
NT-pro BNP, hs-CRP	X					X		X	
Cardiac biomarkers^b^		X				X			
Cardiac function (echocardiography)	X								X
Electrocardiogram and HRV		X				X			
QOL and symptom questionnaire^c^		X						X	
Credibility and AES		X				X			
Blinding test		X				X			
Acupuncture treatment		X	X	X	X	X			

*Abbreviations*: *AES* acupuncture expectancy scale, *D5* date of discharge, hs-*CRP* high-sensitivity C-reactive protein, *HRV* heart rate variability, *NT-pro BNP* N-terminal pro b-type natriuretic peptide, *NYHA* New York Heart Association, *QOL* quality of life, *V1* 7 days post-discharge ±5 days, *V2* 28 days post-discharge ±7 days, *V3* 180-days post discharge ±14 days

^a^Routine blood test: complete blood cell count and different counts (CBC, DC), blood urea nitrogen (BUN), creatinine, electrolyte (Na, K, Cl), total bilirubin, glucose, total protein, albumin, aspartate aminotransferase (AST), alanine aminotransferase (ALT)

^b^Cardiac biomarkers: atrial natriuretic peptide (ANP), ST2, catecholamine, vasoactive intestinal peptide (VIP), interleukin 6 (IL-6), vascular adhesion protein-1 (VAP-1)

^c^QOL and symptom questionnaire: EuroQol five dimensions questionnaire (EQ-5D), Minnesota Living with Heart Failure Questionnaire (MLHFQ), pattern identification questionnaire according traditional East Asian medicine theory

#### Treatment group intervention (PSD with a real needle)

For the active treatment group, bilateral PC5, PC6, ST36, ST37, KI3, and SP6 will be stimulated 20 min per day for 5 days using disposable, sterile needles inserted to a depth of 1.5 ± 0.5 cm, and connected to an electrical stimulator (ES-160, ITO, Japan) with a frequency of 2 Hz. Acupuncture points were selected according to experimental/clinical articles, classic acupuncture and meridian theory, clinical experience, and consensus of the Korean Medicine Doctor (KMD) [15–23]. PC5, PC6, ST36, and ST37 are already known to be effective in cardiac disease [15, 16]. As our primary outcome is the total diuretic dose, acupuncture points that have the effect of decreasing diuresis and edema (KI3, SP6) were also selected. Other detailed information of the acupuncture treatment regimen is described in the STandards for Reporting Interventions in Clinical Trials of Acupuncture (STRICTA) checklist [35] (Table 2).

**Table 2:** Details of acupuncture treatments based on the STRICTA 2010 checklist

Item		Detail
1. Acupuncture rationale	1a) Style of acupuncture	Electroacupuncture based on traditional Korean medicine theory
	1b) Reasoning for treatment provided, based on historical context, literature sources, and/or consensus methods, with references where appropriate	Based on consensus of the KMD, clinical experience, traditional acupuncture, meridian theory, and experimental/clinical articles
	1c) Extent to which treatment was varied	All participants will receive standardized treatment
2. Details of needling	2a) Number of needle insertions per subject per session	12
	2b) Names of points used	bilateral PC5, PC6, ST36, ST37, KI3, and SP6
	2c) Depth of insertion, based on a specified unit of measurement	1.5 ± 0.5 cm
	2d) Response sought	De-qi
	2e) Needle stimulation :	Electrical stimulation with a frequency of 2 Hz, 400 μs per stimulation (ES-160, ITO, Japan)
	2f) Needle retention time	20 min
	2 g) Needle type	Park Sham Device (PSD) with a real needle; 0.20 mm × 40 mm sterilized stainless steel needle (Park Sham Device, Acuprime, Exeter, UK).
3. Treatment regimen	3a) Number of treatment sessions	5 sessions
	3b) Frequency and duration of treatment sessions	1 sessions per day for 5 days
4. Other components of treatment	4a) Details of other interventions administered to the acupuncture group	Conventional Western medicine management according to guideline: diuretics, nitroglycerin, angiotensin-converting enzyme inhibitors, angiotensin receptor blockers, beta blockers, aldosterone antagonists, ivabradine
	4b) Setting and context of treatment, including instructions to practitioners, and information and explanations to patients	The study will be conducted at the Kyung Hee University Medical Center. All patients will be admitted to the cardiac ward for treatment of acute heart failure. All information except patient allocated group will be provided to participants.
5. Practitioner background	5) Description of participating acupuncturists	Licensed KMDs with at least 2 years of clinical practice. The practitioners have studied acupuncture for more than 8 years and graduated from the University of Korean Medicine. Standardized operation procedures were written for practitioners to ensure identical treatments.
6. Control interventions	6a) Rationale for the control or comparator in the context of the research question, with sources that justify this choice	Based on consensus of the KMD, clinical experience, traditional acupuncture and meridian theory, and experimental/clinical articles
	6b) Precise description of the control or comparator. If sham acupuncture or any other type of acupuncture-like control is used, provide details as for Items 1 to 3 above.	For the control group, bilateral LI6, LI7, GB37, GB39, BL58 and BL59 will be stimulated 20 min per day for 5 days using PSD with a sham needle (0.25 mm × 40 mm sterilized stainless steel needle) that cannot penetrate the skin. It will be connected to an electrical stimulator without an electrical current.

*Abbreviations*: *KMD* Korean Medicine Doctor, *PSD* park sham device

#### Control group (PSD with a sham needle)

PSD is a validated sham acupuncture device composed of two tubes (Park tube, guide tube) that support the sham needle when penetrating the skin, and a sham needle indistinguishable from a real needle that cannot penetrate the skin [29, 36]. Double-sided tape will fasten the silicon base of the PSD. For the control group, bilateral LI6, LI7, GB37, GB39, BL58, and BL59 will be stimulated 20 min per day for consecutive 5 days using PSD with a sham needle connected to an electrical stimulator (ES-160, ITO, Japan) without electricity. LI6, LI7, GB37, and GB39 are known to be ineffective in cardiac disease [15, 16]. BL58 and BL59 do not decrease diuresis and edema and were selected through consensus of the KMDs. Other detailed information of the acupuncture treatment is described in the STRICTA checklist [35] (Table 2).

#### Co-interventions

The participants will be treated according to Western medicine acute HF management guidelines [2, 37]; diuretics, nitroglycerin, angiotensin-converting enzyme inhibitor, angiotensin receptor blockers, beta blockers, aldosterone antagonists, or ivabradine will be used according to the status of the participant. Other TEAM interventions that can affect results such as herbal medicine, moxibustion, transcutaneous electrical nerve stimulation, or pharmacopuncture will not be allowed.

### Outcome measurements: primary endpoint

A detailed outcome measurement schedule is presented in Table 1. The primary outcome of this trial is the cumulative diuretic dose administered from the date of admission to the date of discharge. The total doses of the treatment and control groups will be compared.

### Outcome measurements: secondary endpoints

Secondary outcomes include both subjective and objective outcomes. Subjective outcomes include symptom improvements measured by the NYHA Functional Classification [38], symptom improvements evaluated by the TEAM pattern identification questionnaire [39–43] and quality of life measured by the EuroQol Five Dimensions Questionnaire (EQ-5D) [44] and the Minnesota Living with Heart Failure Questionnaire (MLHFQ) [45]. Objective outcomes include routine blood tests (renal function and electrolyte imbalance), cardiac biomarkers, NT-pro BNP, hs-CRP, cardiac function measured by echocardiography, HRV, and MACE.

#### Heart failure symptom improvement (NYHA)

The NYHA Functional Classification will be used to assess symptoms of improvement [38]. It categorizes the patient into one of four levels according to limitations during physical activity.

Class I: *“No limitation of physical activity. Ordinary physical activity does not cause undue fatigue, palpitation, dyspnea (shortness of breath).”*
Class II: *“Slight limitation of physical activity. Comfortable at rest. Ordinary physical activity results in fatigue, palpitation, dyspnea (shortness of breath).”*
Class III: *“Marked limitation of physical activity. Comfortable at rest. Less than ordinary activity causes fatigue, palpitation, or dyspnea.”*
Class IV: *“Unable to carry on any physical activity without discomfort. Symptoms of heart failure at rest. If any physical activity is undertaken, discomfort increases.”*

#### Pattern identification (syndrome differentiation) questionnaire

A pattern identification questionnaire according to TEAM theory will be assessed on Day 1 and Visit 2. Food retention (accumulation) (食積) [40], phlegm (痰飮) [39], blood stasis (瘀血) [41], seven-emotion impairment (七情) [43], and fatigue due to overexertion (勞倦) [42] will be diagnosed by KMDs with a validated pattern identification questionnaire. Each questionnaire has a total score and a cut-off value. The change in value from Day 1 to Visit 2 will be compared. The proportion of participants who have certain TEAM syndrome will also be compared.

#### Quality of life

EQ-5D is a self-reported standardized and validated questionnaire that assess QOL using general questions related to health status [44]. We will use the validated Korean version of EQ-5D [46]. MLHFQ is a HF-specific QOL questionnaire [45]. It sensitively assesses change before and after treatment. The changes in value from the baseline of the two groups will be compared.

#### Routine blood tests

Routine blood tests will be performed to evaluate renal function, electrolyte imbalance, and safety. A complete blood cell count, blood urea nitrogen, creatinine, electrolytes (Na, K, Cl), total bilirubin, glucose, total protein, albumin, aspartate aminotransferase, and alanine aminotransferase will be measured.

#### Cardiac biomarkers

Atrial natriuretic peptide, ST2, catecholamine, vasoactive intestinal peptide, IL-6, and vascular adhesion protein-1 will be measured. These cardiac biomarkers were selected according to a consensus among the research team members and previous research [47–55]. The changes in value from the baseline of the two groups will be compared.

#### NT-pro BNP

Both BNP and NT-pro BNP are useful natriuretic peptides that can diagnose acute decompensated HF [56] and predict prognosis [57]. We adopted NT-pro BNP because it has greater prognostic value than BNP [58]. The group with an NT-pro BNP level higher than the median has increased mortality compared to the group with levels lower than median. The change in value of NT-pro BNP from the baseline will be compared.

#### hs-CRP

In HF, chronic inflammation status is related to the pathophysiology of the disease [59]. Patients with more severe HF symptoms have a higher hs-CRP level [60]. On screening and discharge days, hs-CRP will be measured. The changes in value from the baseline between the two groups will be compared.

#### Cardiac function

Echocardiography is a helpful, non-invasive assessment tool of ventricular function and hemodynamics in HF patients. Ejection fraction (%), the ratio between early mitral inflow velocity and mitral annular early diastolic velocity (E/e’ ratio), left ventricular fractional shortening (%), left ventricular end diastolic diameter (mm), and left ventricular end systolic diameter (mm) will be evaluated by echocardiography to assess cardiac function. Follow-up echocardiography will be assessed 180 days after discharge. The changes in value from the baseline between the two groups will be compared.

#### HRV

HRV reflects ANS status [12]. As sympathetic dominance is an important pathophysiology of HF, HRV is a known important prognostic factor of HF [13, 14]. A Holter monitoring device will measure 24-h heart rate variability. The changes in value from baseline between the two groups will be compared.

#### MACE

Mortality due to heart and kidney problems and the readmission rate between the two groups will be compared until 180 days after discharge.

#### Safety and adverse event outcomes

An adverse event refers to any medical event related to the use of intervention in human clinical trials, whether or not it is considered intervention-related. All adverse events will be evaluated and recorded on every visit by the clinical research coordinator and physicians.

### Sample size calculation

The primary outcome of our study is the total diuretic doses of the treatment and control groups during hospitalization. In a previous study about the effect of the adenosine A1-receptor antagonist (rolofylline) in acute decompensated HF and renal impairment patients, the mean and standard deviation of total diuretic doses were reported [25]. In the trial, doses of 0, 2.5, 15, 30, and 60 mg of rolofylline were used. Our trial offers conventional treatments to both groups and compares the diuretic doses of the real acupuncture and sham acupuncture groups. Therefore, through consensus of the research team members, we assume the effect of real and sham acupuncture on the diuretics doses will be similar to 15 mg and 30 mg rolofylline, respectively. In the study, the cumulative dose of furosemide usage during hospitalization in the group receiving 30 mg of rolofylline was 223 ± 26 mg. In the group receiving 15 mg, it was reported to be 256 ± 39 mg. In a superiority test, with 80% of power (1-β = 0.8) and 5% of significance level (α = 0.05) in a two-sided *t*-test, calculated sample size of each group was 17 participants. After determining that the dropout rate will be approximately 20%, the sample size of each group was increased to 22; and the total sample size was 44. Sample size was calculated using G-power 3.1 software (Heinrich-Heine-Universität, Düsseldorf, Germany).

### Statistical analysis

The primary outcome of our study is the cumulative amount of diuretic administered to each group. After a normality test, the total amount will be compared by an independent *t*-test (parametric) or a Mann-Whitney U test (non-parametric) according to the normality of the data. If the baseline characteristic is significantly different between the two groups, an analysis of covariance (ANCOVA) will be used to adjust the baseline difference. In baseline characteristics and secondary outcome analysis, binary outcomes will be compared by a chi-square test or Fisher’s exact test. Continuous outcomes will be compared by an independent *t*-test (parametric) or Mann-Whitney U test (non-parametric). MACE will be also be analyzed using the survival analysis method. Statistical analysis will be performed using the R package by a blinded statistician. A 5% significance level will be used. Missing values will be replaced by the last observation carried forward method. If there are more than 20% of drop out participants, sensitivity analysis will be conducted including the data.

### Quality control

Acupuncture treatment sessions will be conducted by KMDs who have at least 2 years of clinical experience and more than 8 years of education in acupuncture and meridian. SOPs will be provided to practitioners to ensure identical treatment procedures. The SOPs will contain the treatment regimen and process. A moderate grade of doctor-patient relationship is allowed. The doctor is allowed to answer the questions of participants except those concerning the treatment group assignment. Researcher meetings will be held regularly to ensure the quality of the clinical trial. Assessor will be educated about evaluation and data collection according to SOPs. Study process will be monitored by human research protections program.

### Data management

Data will be entered by independent researcher. Data will be stored on password-protected computers. Data manager who is independent from competing interests can only access to the data.

### Ethics approval and registration

This study was planned in compliance with the Korean Good Practice Guidelines and the Helsinki Declaration to protect the rights of participants. This study was approved by the Institutional Review Board of Kyung Hee Medical Center (KMC IRB 1601–01-A1). An initial version of this protocol was approved by KMCIRB on March 23, 2016. Written informed consent will be obtained from all participants. The participants will be informed about the risks, benefits, responsibilities, alternatives, and rights during the study. This study has been registered with the Clinical Research Information Service (CRIS), Republic of Korea, KCT0002249 (registered February 14, 2017). If protocol is modified, it will be reported to IRB and announced to the participants and investigators. Personal information will be collected by clinical research coordinators and stored password-protected computer to protect confidentiality.

## Discussion

The purpose of our trial is to assess the efficacy and safety of electroacupuncture compared with sham electroacupuncture treatment in acute decompensated HF patients. Several implications for future acupuncture clinical trials suggested by a previous systematic review were considered for our protocol [24]. The primary outcome of our trial is the total dose of diuretic administered during hospitalization. As excessive diuretics cause electrolyte disorder, which is a risk factor of cardiovascular events [61], decreased diuretic use induced due to acupuncture treatment will prove therapeutic value of acupuncture in HF treatment. Subjective secondary outcomes of our trial include NYHA Functional Classification, the TEAM pattern identification questionnaire [39–43], and QOL questionnaires such as EQ-5D and MLHFQ. Objective secondary outcomes include routine blood tests, cardiac biomarkers, NT-pro BNP, hs-CRP, cardiac function, HRV, and MACE. As we will investigate TEAM pattern identification, we can report distribution of TEAM syndrome in acute HF patients. This study will provide fundamental data for future acupuncture and herbal medicine clinical trials of HF. If possible, subgroup analysis that identifies a response to acupuncture treatment classified by TEAM syndrome differentiation will be conducted. We will also investigate the expectations and credibility of acupuncture, as they are known to affect the therapeutic effect of acupuncture [62, 63]. The efficacy paradox is a well-known phenomenon in the acupuncture clinical trial using a placebo or sham acupuncture. As the effect size of the sham acupuncture treatment is higher than that of placebo medicine, many acupuncture trials have failed to prove the effects of acupuncture compared to placebo acupuncture [64]. Therefore, we tried to decrease the anticipated effect size of the control group. To decrease the effect size of the control group, we adopted PSD in the control group that cannot penetrate skin, selected different acupuncture points for the treatment group, and will use an electric stimulator that does not deliver electricity during control group treatments.

To summarize, our clinical trial protocol adopted several implications for future study from previous reviews that can offer new and sound evidence of electroacupuncture treatment in acute decompensated HF patients.

### Trial status

This trial is currently recruiting participants. Recruitment began on March 26, 2016. We expect recruitment to be complete by the end of 2019.

## Abbreviations

ANS: Autonomic nervous system
BI: Blinding index
BNP: B-type natriuretic peptide
EQ-5D: EuroQol five dimensions questionnaire
HF: Heart failure
HRV: Heart rate variability
hs-CRP: High-sensitivity C-reactive protein
IRB: Institutional review board
KHMC: Kyung hee medical center
KMD: Korean medicine doctor
MACE: Major adverse cardiac event
MLHFQ: Minnesota living with heart failure questionnaire
NT-pro BNP: N-terminal pro b-type natriuretic peptide
NYHA: New York heart association
PSD: Park sham device
QOL: Quality of life
SOP: Standard operating procedure
STRICTA: STandards for reporting interventions in clinical trials of acupuncture
TEAM: Traditional east asian medicine
V1: Visit 1
V2: Visit 2
V3: Visit 3

## References

[1] Hunt SA (2005). American College of Cardiology, American Heart Association Task Force on practice guidelines (Writing Committee to update the 2001 guidelines for the evaluation and Management of Heart Failure). ACC/AHA 2005 guideline update for the diagnosis and management of chronic heart failure in the adult: a report of the American College of Cardiology/American Heart Association Task Force on practice guidelines (Writing Committee to update the 2001 guidelines for the evaluation and Management of Heart Failure). J Am Coll Cardiol.

[2] Yancy CW, Jessup M, Bozkurt B, Butler J, Casey DE, Writing Committee Members (2016). 2016 ACC/AHA/HFSA Focused Update on New Pharmacological Therapy for Heart Failure: An Update of the 2013 ACCF/AHA Guideline for the Management of Heart Failure: A Report of the American College of Cardiology/American Heart Association Task Force on Clinical Practice Guidelines and the Heart Failure Society of America. Circulation.

[3] Roger VL (2013). Epidemiology of heart failure. Circ Res.

[4] Ho KK, Pinsky JL, Kannel WB, Levy D (1993). The epidemiology of heart failure: the Framingham study. J Am Coll Cardiol.

[5] MacIntyre K, Capewell S, Stewart S, Chalmers JW, Boyd J, Finlayson A (2000). Evidence of improving prognosis in heart failure: trends in case fatality in 66 547 patients hospitalized between 1986 and 1995. Circulation.

[6] Mosterd A, Cost B, Hoes AW, de Bruijne MC, Deckers JW, Hofman A (2001). The prognosis of heart failure in the general population: the Rotterdam study. Eur Heart J.

[7] Mao J, Hou Y, Shang H, Wang H, Wang X, Zhao Y (2009). Study on the evaluation of the clinical effects of traditional chinese medicine in heart failure by complex intervention: protocol of SECETCM-HF. Trials.

[8] Li X, Zhang J, Huang J, Ma A, Yang J, Li W (2013). A multicenter, randomized, double-blind, parallel-group, placebo-controlled study of the effects of qili qiangxin capsules in patients with chronic heart failure. J Am Coll Cardiol.

[9] Tang WHW, Huang Y (2013). Cardiotonic modulation in heart failure: insights from traditional Chinese medicine. J Am Coll Cardiol.

[10] Xian S, Yang Z, Lee J, Jiang Z, Ye X, Luo L (2016). A randomized, double-blind, multicenter, placebo-controlled clinical study on the efficacy and safety of Shenmai injection in patients with chronic heart failure. J Ethnopharmacol.

[11] Florea VG, Cohn JN (2014). The autonomic nervous system and heart failure. Circ Res.

[12] Lee S, Lee MS, Choi J-Y, Lee S-W, Jeong S-Y, Ernst E (2010). Acupuncture and heart rate variability: a systematic review. Auton Neurosci Basic Clin.

[13] Sandercock GRH, Brodie DA (2006). The role of heart rate variability in prognosis for different modes of death in chronic heart failure. Pacing Clin Electrophysiol PACE.

[14] Chattipakorn N, Incharoen T, Kanlop N, Chattipakorn S (2007). Heart rate variability in myocardial infarction and heart failure. Int J Cardiol.

[15] Longhurst J (2013). Acupuncture’s cardiovascular actions: a mechanistic perspective. Med Acupunct.

[16] Painovich J, Longhurst J (2015). Integrating acupuncture into the cardiology clinic: can it play a role?. Sheng Li Xue Bao.

[17] Middlekauff HR, Hui K, Yu JL, Hamilton MA, Fonarow GC, Moriguchi J. Acupuncture inhibits sympathetic activation during mental stress in advanced heart failure patients. J Card Fail. 2002;8(6):399–406. 10.1054/jcaf.2002.129656 12528093

[18] Li J-B (2003). Clinical research on the effect of acupuncture on the left ventricular contractin function in symptomless cardiac failure patients. World J Acupunct-MoxibustionShi Jie Zhen Jiu Za Zhi.

[19] Kristen AV, Schuhmacher B, Strych K, Lossnitzer D, Friederich HC, Hilbel T (2010). Acupuncture improves exercise tolerance of patients with heart failure: a placebo-controlled pilot study. Heart Br Card Soc.

[20] Li ZY, Lao MX, Pan QJ (2012). Effect of acupuncture on Hemodynamics and cardiac function in patients with chronic heart failure. Shanghai J Acu-Mox.

[21] Liu D, Zou J, Luo X, Zhang B (2014). Curative observation of adopting high frequency electro-acupuncture at Neiguan to treat heart failure complicated by acute myocardial infarction. J Sichuan Tradit Chin Med.

[22] Xiao QS, Zhang B, Ma MY, Deng MH, Yang YZ (2014). Effect of acupuncture on acute left heart failure by PiCCO technique. Chin J Integr Tradit West Med.

[23] Qu XX, Yang KY, Shen Y (2015). Effects of acupuncture about heart rate variability on chronic heart failure patients. Shaanxi J Tradit Chin Med.

[24] Lee H, Kim T-H, Leem J (2016). Acupuncture for heart failure: a systematic review of clinical studies. Int J Cardiol.

[25] Givertz MM, Massie BM, Fields TK, Pearson LL, Dittrich HC (2007). CKI-201 and CKI-202 investigators. The effects of KW-3902, an adenosine A1-receptor antagonist,on diuresis and renal function in patients with acute decompensated heart failure and renal impairment or diuretic resistance. J Am Coll Cardiol.

[26] Cotter G, Dittrich HC, Weatherley BD, Bloomfield DM, O’Connor CM, Metra M (2008). The PROTECT pilot study: a randomized, placebo-controlled, dose-finding study of the adenosine A1 receptor antagonist rolofylline in patients with acute heart failure and renal impairment. J Card Fail.

[27] Weatherley BD, Cotter G, Dittrich HC, DeLucca P, Mansoor GA, Bloomfield DM (2010). Design and rationale of the PROTECT study: a placebo-controlled randomized study of the selective A1 adenosine receptor antagonist rolofylline for patients hospitalized with acute decompensated heart failure and volume overload to assess treatment effect on congestion and renal function. J Card Fail.

[28] Voors AA, Dittrich HC, Massie BM, Delucca P, Mansoor GA, Metra M (2011). Effects of the adenosine A1 receptor antagonist rolofylline on renal function in patients with acute heart failure and renal dysfunction: results from PROTECT (placebo-controlled randomized study of the selective adenosine A1 receptor antagonist Rolofylline for patients hospitalized with acute Decompensated heart failure and volume overload to assess treatment effect on congestion and renal function). J Am Coll Cardiol.

[29] Park J, White A, Stevinson C, Ernst E, James M (2002). Validating a new non-penetrating sham acupuncture device: two randomised controlled trials. Acupunct. Med J Br Med Acupunct Soc.

[30] Chan A-W, Tetzlaff JM, Altman DG, Laupacis A, Gøtzsche PC, Krleža-Jerić K (2013). SPIRIT 2013 statement: defining standard protocol items for clinical trials. Ann Intern Med.

[31] Bang H, Ni L, Davis CE (2004). Assessment of blinding in clinical trials. Control Clin Trials.

[32] Lee H, Bang H, Kim Y, Park J, Lee S, Lee H (2011). Non-penetrating sham needle, is it an adequate sham control in acupuncture research? Complement. Ther Med.

[33] Vincent C (1990). Credibility assessment in trials of acupuncture. Complement Med Res.

[34] Kim Y-J, Lee I-S, Kim H-S, Lee H, Park H-J, Lee H (2014). Validation of the Korean version of the acupuncture expectancy scale. Acupunct. Med J Br Med Acupunct Soc.

[35] MacPherson H, Altman DG, Hammerschlag R, Li Y, Wu T, White A (2010). Revised STandards for reporting interventions in clinical trials of acupuncture (STRICTA): extending the CONSORT statement. Acupunct. Med J Br Med Acupunct Soc.

[36] Leem J, Park J, Han G, Eun S, Makary MM, Park K (2016). Evaluating validity of various acupuncture device types: a random sequence clinical trial. BMC Complement Altern Med.

[37] Ponikowski P, Voors AA, Anker SD, Bueno H, Cleland JGF, Coats AJS (2016). 2016 ESC guidelines for the diagnosis and treatment of acute and chronic heart failure: the Task Force for the diagnosis and treatment of acute and chronic heart failure of the European Society of Cardiology (ESC). Developed with the special contribution of the heart failure association (HFA) of the ESC. Eur. J. Heart Fail.

[38] The Criteria Committee of the New York Heart Association (1994). Nomenclature and criteria for diagnosis of diseases of the heart and great vessels.

[39] Park Y-J, Park J-S, Kim M-Y, Park Y-B (2011). Development of a valid and reliable phlegm pattern questionnaire. J Altern Complement Med N Y N.

[40] Park Y-J, Lim J-S, Park Y-B (2013). Development of a valid and reliable food retention questionnaire. Eur J Integr Med.

[41] Park Y-J, Yang D-H, Lee J-M, Park Y-B (2013). Development of a valid and reliable blood stasis questionnaire and its relationship to heart rate variability. Complement Ther Med.

[42] Yoon K-J, Park Y-B, Park Y-J, Kim M-Y (2015). Development and validation of a Lao Juan (劳倦) questionnaire. Chin J Integr Med.

[43] Lee B-H, Kim M-Y, Park Y-B, Park Y-J (2016). Development of a valid and reliable seven emotions impairment questionnaire and assessment of its predictability for phlegm and blood stasis. J Tradit Chin Med.

[44] Dolan P (1997). Modeling valuations for EuroQol health states. Med Care.

[45] Oldridge NB (1997). Outcome assessment in cardiac rehabilitation. Health-related quality of life and economic evaluation. J Cardpulm Rehabil.

[46] Park D-J, Kang J-H, Lee J-W, Lee K-E, Wen L, Kim T-J (2013). Cross-cultural adaptation of the Korean version of the Boston carpal tunnel questionnaire: its clinical evaluation in patients with carpal tunnel syndrome following local corticosteroid injection. J Korean Med Sci.

[47] Kubo SH, Rector TS, Williams RE, Heifetz SM, Bank AJ (1991). Endothelium-dependent vasodilation is attenuated in patients with heart failure. Circulation.

[48] Hirooka Y, Imaizumi T, Tagawa T, Shiramoto M, Endo T, Ando S (1994). Effects of L-arginine on impaired acetylcholine-induced and ischemic vasodilation of the forearm in patients with heart failure. Circulation.

[49] Tan LB, Jalil JE, Pick R, Janicki JS, Weber KT (1991). Cardiac myocyte necrosis induced by angiotensin II. Circ Res.

[50] Wilkins MR, Redondo J, Brown LA (1997). The natriuretic-peptide family. Lancet Lond Engl.

[51] Mann DL (2001). Interleukin-6 and viral myocarditis: the yin-Yang of cardiac innate immune responses. J Mol Cell Cardiol.

[52] Bayes-Genis A, Pascual-Figal D, Januzzi JL, Maisel A, Casas T, Valdés Chávarri M (2010). Soluble ST2 monitoring provides additional risk stratification for outpatients with decompensated heart failure. Rev Esp Cardiol.

[53] Shah RV, Januzzi JL (2010). ST2: a novel remodeling biomarker in acute and chronic heart failure. Curr Heart Fail Rep.

[54] Jaakkola K, Jalkanen S, Kaunismäki K, Vänttinen E, Saukko P, Alanen K (2000). Vascular adhesion protein-1, intercellular adhesion molecule-1 and P-selectin mediate leukocyte binding to ischemic heart in humans. J Am Coll Cardiol.

[55] Umetsu Y, Tenno T, Goda N, Shirakawa M, Ikegami T, Hiroaki H (1814). Structural difference of vasoactive intestinal peptide in two distinct membrane-mimicking environments. Biochim Biophys Acta.

[56] Januzzi JL, van Kimmenade R, Lainchbury J, Bayes-Genis A, Ordonez-Llanos J, Santalo-Bel M (2006). NT-proBNP testing for diagnosis and short-term prognosis in acute destabilized heart failure: an international pooled analysis of 1256 patients: the international collaborative of NT-proBNP study. Eur Heart J.

[57] Chen AA, Wood MJ, Krauser DG, Baggish AL, Tung R, Anwaruddin S (2006). NT-proBNP levels, echocardiographic findings, and outcomes in breathless patients: results from the ProBNP investigation of Dyspnoea in the emergency department (PRIDE) echocardiographic substudy. Eur Heart J.

[58] Waldo SW, Beede J, Isakson S, Villard-Saussine S, Fareh J, Clopton P (2008). Pro-B-type natriuretic peptide levels in acute decompensated heart failure. J Am Coll Cardiol.

[59] Matsumori A (2004). Anti-inflammatory therapy for heart failure. Curr Opin Pharmacol.

[60] Windram JD, Loh PH, Rigby AS, Hanning I, Clark AL, Cleland JGF (2007). Relationship of high-sensitivity C-reactive protein to prognosis and other prognostic markers in outpatients with heart failure. Am Heart J.

[61] Faris RF, Flather M, Purcell H, Poole-Wilson PA, Coats AJS. Diuretics for heart failure. Cochrane Database Syst Rev. 2012;5;(2):CD003838. doi:10.1002/14651858.CD003838.pub3. 10.1002/14651858.CD003838.pub3 22336795

[62] Linde K, Witt CM, Streng A, Weidenhammer W, Wagenpfeil S, Brinkhaus B (2007). The impact of patient expectations on outcomes in four randomized controlled trials of acupuncture in patients with chronic pain. Pain.

[63] Zheng H, Huang W, Li J, Zheng Q, Li Y, Chang X (2015). Association of pre- and post-treatment expectations with improvements after acupuncture in patients with migraine. Acupunct. Med J Br Med Acupunct Soc.

[64] Linde K, Niemann K, Schneider A, Meissner K (2010). How large are the nonspecific effects of acupuncture? A meta-analysis of randomized controlled trials. BMC Med.

